# Bold strides towards the elimination of *gambiense* human African trypanosomiasis (gHAT) as a public health problem—A case study of Angola

**DOI:** 10.1371/journal.pntd.0012847

**Published:** 2025-02-12

**Authors:** Johnson O. Ouma, Simon Kayembe, Paul R. Bessell, Don Paul Makana, Amadeu D. C. P. Dala, Luis Baião Peliganga, Joseph M. Ndung’u, Constantina P. F. Machado

**Affiliations:** 1 Foundation for Innovative New Diagnostics (FIND)—Kenya, Nairobi, Kenya; 2 Department of Tropical Medicine, University of Kinshasa, in Kinshasa, Democratic Republic of Congo; 3 Institute for the Combat and Control of Trypanosomiases (ICCT), Ministry of Health, Luanda, Angola; 4 Office of the Deputy Governor, Cuanza Norte Province, N’dalatando, Angola; University of Washington, UNITED STATES OF AMERICA

## Abstract

**Background:**

The chronic form of human African trypanosomiasis (HAT) caused by *Trypanosoma brucei gambiense* and commonly referred to as *gambiense*-HAT (gHAT) is endemic in 7 of Angola’s 18 provinces. Major epidemics of the disease occurred in the country in the 1920s to 1940s and 1990s –mid 2000s, and current estimates are that up to a third of the country’s population is at risk of infection. Whereas gHAT was first reported in Angola in 1871, control efforts did not begin until 30 years later in 1901. This case study describes the history of gHAT in Angola, outlines the policies and strategies used in its control, and the intensification efforts being made to accelerate progress towards elimination. Furthermore, it highlights factors that have contributed to recurrent outbreaks of gHAT in the country and key achievements in the push towards elimination.

**Methods:**

Literature review was conducted using online databases such as PubMed, Google Scholar, Google, WHO HAT data repository, and the African Union Inter African Bureau for Animal Resources (AU-IBAR), International Scientific Council for Trypanosomiasis Research and Control (ISCTRC) conference proceedings. Data/information not found in these databases was availed through personal communication with colleagues from *Instituto de Combate e Controlo das Tripanossomiases* (ICCT). The search of databases was conducted using the following terms: “human African trypanosomiasis (HAT) control/elimination in Angola,” “sleeping sickness/HAT control in Angola,” “HAT epidemics in Angola.”

**Results and conclusion:**

Overall, the interventions put in place over the years have led to significant reduction in the number of new HAT cases reported annually, from an average of 3,496 (between 1990 and 2006) to an average of 56 cases between 2016 and 2023. This has renewed the hope of achieving elimination of gHAT as a public health problem by 2030.

## Author summary

Human African trypanosomiasis (HAT), also known as sleeping sickness, is a disease caused by trypanosome parasites transmitted by tsetse flies. HAT presents in 2 forms (chronic or acute) depending on the subspecies of the parasite causing the disease. Angola has the chronic form of HAT and the areas and populations at-risk of HAT are large, resulting in the country experiencing very large epidemics in the past. Through coordinated and sustained control operations, largely supported by the national government, the number of HAT cases reported annually has been bought under control, and the country is progressing towards HAT elimination.

This case study is important because it highlights the strategies and policies that have led to sustainable control of HAT in Angola, and key lessons that other national HAT control programs could borrow to achieve a more sustainable and impactful disease control. Among key factors that contributed to sustainable HAT control in Angola and which other national programs could adopt are the following: (1) strong political will and government commitment to fund most HAT control activities; (2) adoption and roll-out of new technologies through the health systems; and (3) partnering with state and non-state actors to sustain control of the disease, particularly in times of internal instability.

## Introduction

**Fig 1:**
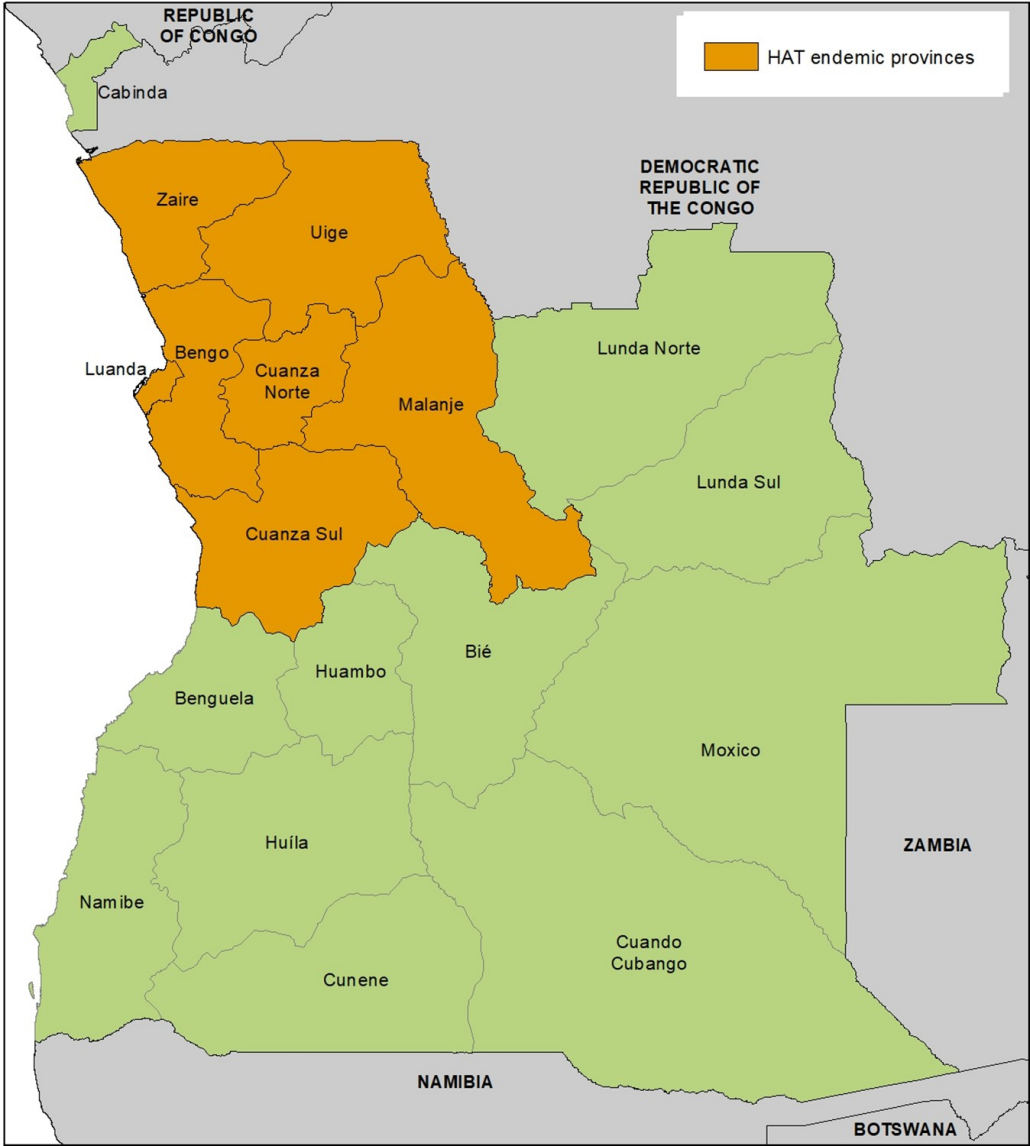
Map of Angola showing all 18 administrative and 7 HAT endemic provinces. Map created with ESRI ArcPro v3.3. Boundary data from GADM (https://gadm.org/license.html).

The Republic of Angola is located in the southwestern region of Africa, bordering Namibia to the south, Democratic Republic of the Congo (DRC) to the north, and Zambia to the east. It borders the Atlantic Ocean to the west. Angola occupies a land area of 1,246,700 km^2^ and has 18 administrative provinces, 7 of which are endemic for human African trypanosomiasis (HAT) (Fig 1).

In 2023, the country’s population was estimated to be 34.1 million people, 45% of whom live in rural areas [1]. It is estimated that one fifth of Angola’s population is at risk of contracting *gambiense* human African trypanosomiasis (gHAT) (sleeping sickness) [2], a chronic form of the disease caused by *Trypanosoma brucei gambiense* (Tbg). Given the large areas and populations at risk of HAT, the country previously experienced major waves of outbreaks and epidemics, particularly in the 1920s to 1940s and 1990s to mid-2000s. The disease, which is transmitted by tsetse flies, was first reported in the Quicama region in the late 19th century and is endemic in the northwestern part of the country [2]. Most of the high-risk areas are found in the provinces of Bengo, Cuanza Norte, Uige, and Zaire, with other provinces such as Cuanza Sul, Malanje, Luanda, and Cabinda being considered low-risk areas [3]. *Glossina fuscipes s*.*l*. and *G*. *palpalis* are the predominant tsetse fly species in the country [4], with *G*.*p*. *palpalis* considered the principal vector of sleeping sickness [5]. The acute form of HAT, caused by *T*. *b*. *rhodesiense* and transmitted by *G*. *morsitans centralis*, was previously reported in the southern province of Cuando Cubango, but is believed to have disappeared from the country, due to movement of human populations to the northern part of Angola during a civil war between 1975 and 2002 [6].

Historically, Angola has been one of the most HAT endemic countries, and by 2002, was reporting more than 3,600 cases per year [7]. Prior to 2002, efforts to control the disease were hampered by an armed conflict that went on for nearly 30 years. Following the signing of a peace agreement in 2002, the country’s Ministry of Health embarked on a program to control the disease, coordinated by *Instituto de Combate e Controlo das Tripanossomiases* (ICCT). Programmatic activities included an aggressive active screening campaign using the card agglutination test for trypanosomiasis (CATT), passive screening in a limited number of diagnostic and treatment facilities, and vector control. These efforts were largely funded by the Angolan government and led to a significant decline in the number of reported HAT cases, from 8,275 in 1997 to only 20 in 2016 [7], the year when the Foundation for Innovative New Diagnostics (FIND) partnered with ICCT.

This case-study reviews the history of gHAT and its control in Angola since 1901, evaluates the policies and main interventions applied over time, including in the containment of outbreaks/epidemics, and highlights factors contributing to recurrent outbreaks of the disease. Partnering with external and internal organizations that have strong influence in the community such as the church (e.g., Caritas de Angola) and nongovernmental organizations in the fight against gHAT is highlighted as one of the key drivers of success in HAT control in Angola [5]. Additionally, this case study describes the strategies being implemented to accelerate progress towards elimination of gHAT as a public health problem and highlights key achievements resulting from the push towards elimination of the disease. The case study outlines the health system that has supported control efforts in recent years and demonstrates strong political commitment and mobilization of resources needed to progress towards HAT elimination.

## Materials and methods

A literature review was conducted using online databases such as PubMed, Google Scholar, Google, WHO HAT data repository (https://apps.who.int/neglected_diseases/ntddata/hat/hat.html), and proceedings of the International Scientific Council for Trypanosomiasis Research and Control (ISCTRC) conference (https://www.au-ibar.org/au-ibar-secretariats/international-scientific-council-trypanosomiasis-research-and-control), a publication of the African Union Inter African Bureau for Animal Resources (AU-IBAR). Data/information not found in these databases was availed through personal communication with colleagues from *Instituto de Combate e Controlo das Tripanossomiases* (ICCT). The search of databases was conducted using the following terms: “human African trypanosomiasis (HAT) control/elimination in Angola,” “sleeping sickness/HAT control in Angola,” “HAT epidemics in Angola.” To understand the local health system that has supported HAT control in Angola, the following search terms were used in the databases outlined above in conjunction with the word Angola: “health policy,” “health system,” “health budget,” “health programs.” It was noted during the literature review that there is a very limited number of published reports on HAT and its control in Angola. Hence, some of the information/data reported in this case study was obtained from ICCT leadership and scientists who are listed as co-authors in this manuscript.

## Results

### History of human African trypanosomiasis and its control in Angola

#### (a) Historical landmarks in gHAT control and initiation of elimination campaigns

**Fig 2:**
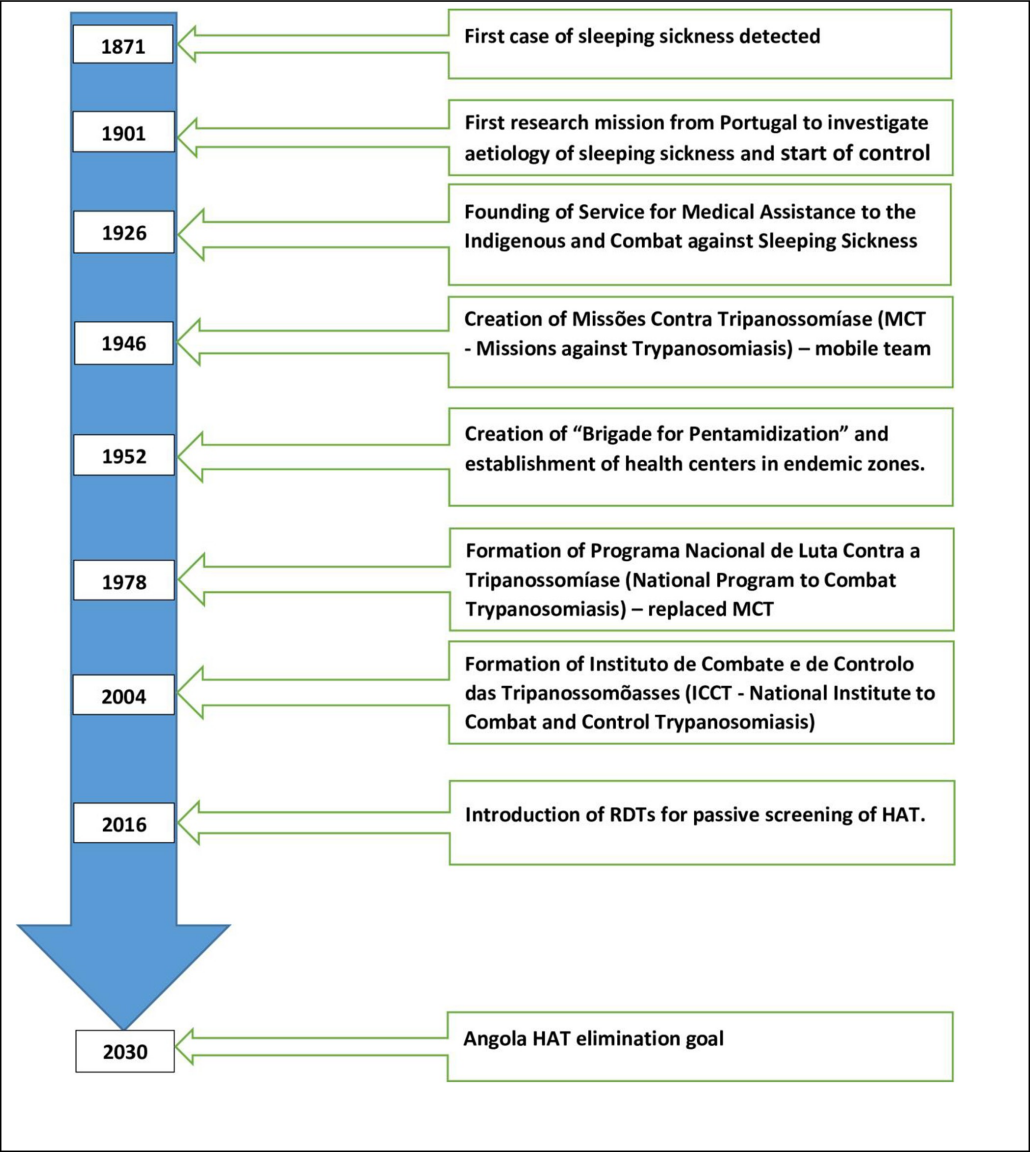
Landmarks in the history of HAT and its control in Angola.

Fig 2 outlines the historical landmarks in HAT control and initiation of elimination campaigns in Angola.

The first landmark was the reporting of the first case of HAT in the Quicama region along River Kwanza, south of Luanda, in the late 19th century [8]. Elsewhere, it was suggested that the first case of sleeping sickness could have been reported in 1871 in Massangano, near Dondo, which was then a key commercial hub [9]. Exploration of the hinterlands of Angola and most of the rural central African region during the colonial era could have altered the existing ecological equilibrium and exposed laborers to bites by tsetse flies infected with human infective trypanosomes, leading to an explosion in the number of sleeping sickness cases [9–11]. Due to limited medical knowledge on the aetiology of sleeping sickness at the time, the then Portuguese colonial administration initiated scientific research on the disease in 1901 [12].

The second landmark was the initiation of HAT control. Whereas the first case of HAT was reported in 1871, the fight against HAT in the country began in 1901, initially using rudimentary methods, under the directorship of Aníbal Bettencourt [8] who was a physician, microbiologist, and parasitologist. During the first quarter of the 1900s, the methods employed to control HAT in Angola included bush clearing, resettlement of populations away from tsetse fly-infested areas, isolation of sleeping sickness patients into isolation camps and treating them with trypanocidal drugs (notably atoxyl) on the basis of clinical diagnosis. However, these campaigns did not yield the desired impact due to limited personnel and funds [13].

The third landmark was the creation of the first specialized unit for HAT control in 1926, a year when sleeping sickness control became a cornerstone of Angola’s program on rural healthcare and development. In that year, the Portuguese colonial administration created the “Service for Medical Assistance to the Indigenous and Combat against Sleeping Sickness” and introduced microscopy to conduct parasitological tests, leading to screening of large portions of the population at risk [12,13]. Infected individuals were treated with atoxyl or tryparsamide. Interestingly, preventive mass treatment with atoxyl was also carried out as an emergency measure to cope with high infection rates of up to 30% in certain areas (e.g., Cuanza). Mass preventative treatment was based on clinical symptoms and/or mere suspicion of infection with sleeping sickness. This mass drug administration (mass atoxylization) campaign was however abandoned in the early 1930s due to ethical concerns [13] and considering the high toxicity of atoxyl. Nevertheless, the proponents of mass atoxylization argued that continuous atoxylization interrupted the transmission cycle between flies and humans by rendering bodies of HAT-infected individuals noninfectious and by protecting healthy individuals against infection for up to 6 months [13].

The fourth stage in the fight against HAT was the establishment of the first specialized mobile teams. In the mid-1940’s, the HAT control unit was reorganized to create MCT (Missões Contra Tripanossomíase/Missions against Trypanosomiasis). MCT consisted of well-trained mobile teams (physicians, nurses, technicians, and support staff) undertaking active case detection and treatment in the field [13] and had administrative and budgetary autonomy [9]. Prior to any active HAT screening event, the teams would mobilize target communities through the local administration to present themselves for screening. The efforts of the mobile teams significantly enhanced HAT surveillance.

The fifth landmark was the initiation of a controversial mass chemotherapy program [12,13]. To strengthen HAT case management, the colonial health authorities created the “Brigade for Pentamidinization” in 1952 and established 26 fixed health sectors (health units, each consisting of a doctor, nurses, and a few small hospitals) in the endemic zones (Zaire, Uige, Bengo, and Kwanza Norte). Additionally, four (4) mobile teams, each comprising of a doctor, nurses, and microscopists, were created. These traveled from one health sector to another, examining people and treating identified HAT cases with pentamidine. Decisions on whether to treat patients for HAT were based on a combination of clinical symptoms and detection of parasites in body fluids by microscopy. Screening was so intensified that in the 1950s, the mobile teams were examining nearly a million thick blood films annually [13]. As was the case during mass atoxylization, it was not only confirmed cases of HAT that received pentamidine treatment—all other persons within the isolation camps also received prophylactic injections of pentamidine. The mass chemoprophylaxis program using pentamidine was implemented until the early 1970s and was reported to have achieved spectacular success in controlling HAT. The number of new sleeping sickness cases, which was reported to have nearly doubled between 1944 and 1949, significantly declined, to only 120 new cases in 1957 [13].

Another key landmark in the history of HAT control in Angola was the establishment of the national HAT (sleeping sickness) control program, as a means of taking more ownership of HAT control activities in the country. Unfortunately, the program did not become fully operational due the impact of an armed civil conflict at the time, which caused displacement of health workers, severe shortage of vehicles and equipment, and destruction of medical facilities dedicated to trypanosomiasis control [8]. Fortunately, with the support of Swedish Agency for International Development (ASDI) and the World Health Organization (WHO), HAT control activities resumed in 1982 and continued until 1992, before being disrupted again by re-emergence of civil conflict resulting from post-electoral disputes. Nevertheless, with support from external partners such as Médecins Sans Frontières (MSF), Caritas, and Norwegian Peoples’ Aid, and signing of the Lusaka Peace Agreement in 1994, HAT control activities (screening and treatment) resumed in some of the endemic zones [8]. The Catholic Church also played a key role in creating an enabling environment for disease control through mediation between the warring parties [5]. Later, the French Cooperation supported the restructuring of the national HAT control program, leading to formation of the Institute for the Combat and Control of Trypanosomiasis (ICCT) in 2004. The ICCT, working under the supervision of the Ministry of Health, is mandated to develop strategies, policies, and actions for control of sleeping sickness and its vector, the tsetse fly. The institute also conducts diagnosis and treatment, as well as follow-up of HAT patients. Additionally, ICCT carries out epidemiological surveillance of HAT in the country and coordinates activities of different partners in the fight against sleeping sickness.

**Fig 3:**
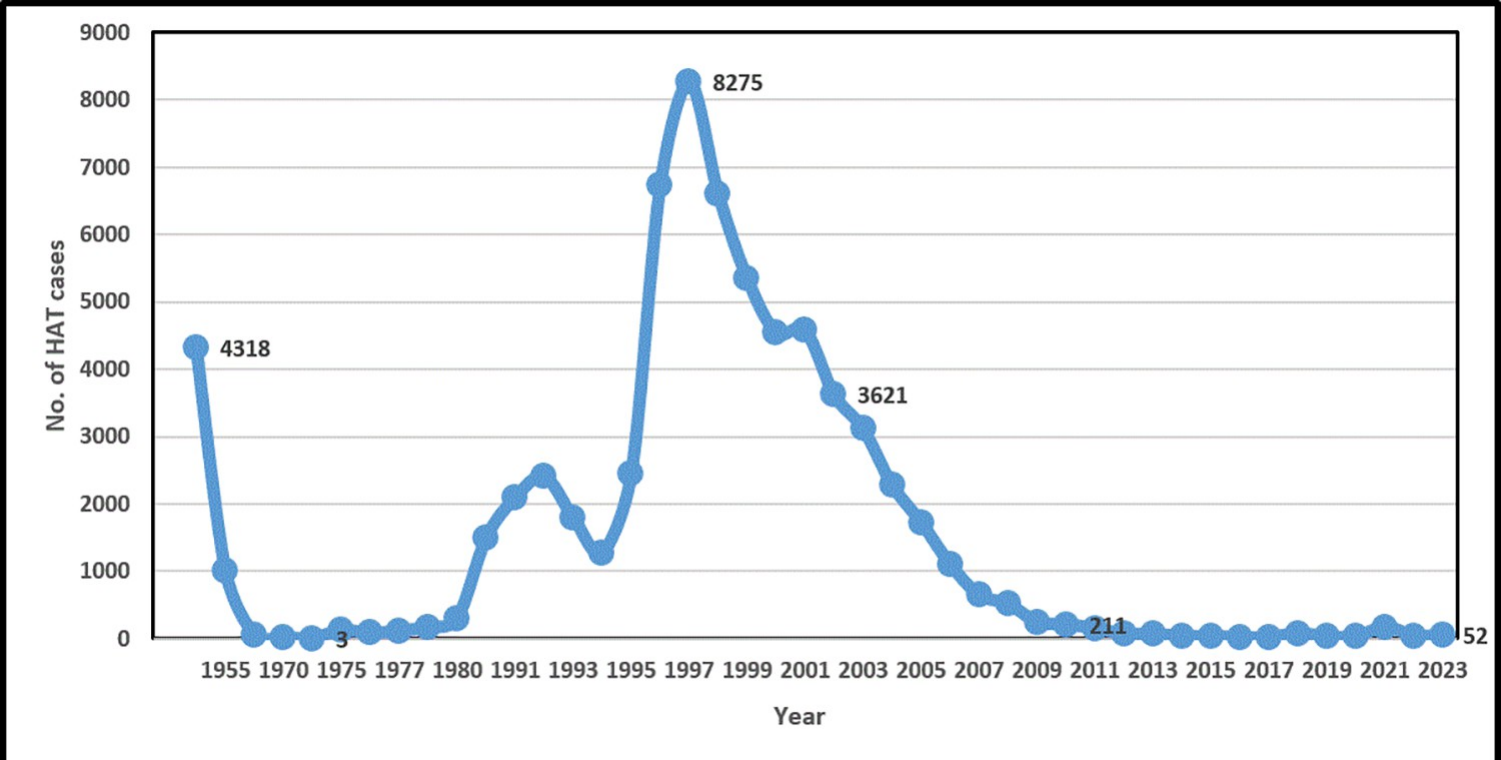
The number of new HAT cases reported in Angola between 1949 and 2023.

In 2012, pharmaceutical companies, donors, endemic countries (Angola included), and nongovernmental organizations came together to sign the London Declaration on neglected tropical diseases (NTDs). The Declaration renewed the commitment of stakeholders to eliminate HAT as a public health problem. Thereafter, the WHO published a roadmap on NTDs that targeted to eliminate HAT as a public health problem by 2020 [14,15]. One of the significant milestones in the control of HAT was the launch of a HAT rapid diagnostic test (RDT) in 2012, coinciding with the London Declaration. The HAT RDT brought with it opportunities for implementation of novel HAT screening strategies that ensured better coverage of the population at risk, thus increasing chances of identifying infected individuals, effectively reducing transmission and making elimination feasible. The first of such strategies was implemented in Uganda from 2013 [16]. In 2014, a collaboration between Angola, the Democratic Republic of Congo (DRC), and Republic of Congo (RoC), sought to adopt novel strategies to eliminate gHAT in the Kongo Central (formerly Bas Congo) transboundary focus [17]. In Angola, the initiative, which included the provinces of Zaire and Cabinda, was subsequently expanded to all the endemic provinces by 2018, further contributing to the steady decrease in new HAT cases reported in Angola (Fig 3). This initiation of the push towards HAT elimination renewed the aspirations and commitment of the country to eliminate HAT, and therefore, was a significant landmark.

#### (b) HAT epidemics/outbreaks and their control

A HAT epidemic that occurred in Angola in the 1920s peaked in 1940, with approximately 9,000 identified cases, dropping to 4,318 cases in 1949 (Fig 3). This epidemic was effectively controlled through active screening by mobile teams, combined with curative and mass prophylactic treatment of populations in the so called “concentration” camps using pentamidine (pentamidinization) [8].

As indicated in section 2(a), mass treatment resulted in significant reduction in the annual number of reported new HAT cases, from 4,318 in 1949 to only 63 new cases in 1960 (Fig 3).

The number of new cases continued to decrease, from 63 in 1960 to 26 in 1970, and in 1974, the year just before Angola gained independence from Portugal, only 3 new HAT cases were reported. This significant reduction in infections was attributed in part to the involvement of the private sector in HAT control activities [13]. In the late 1970s, HAT was no longer considered a pressing public health problem in Angola, and it plummeted on the priority list in the National Health Service’s limited budget.

The number of new HAT cases reported in Angola annually had been on an upward trajectory from 1990 to 1992 (Fig 3). The number dropped from 2,406 in 1992 to 1,796 and 1,274 in 1993 and 1994, respectively, likely due to disruption of surveillance activities resulting from a flare up of post-electoral violence in 1992. The strategies used to control the HAT epidemics of 1990s were case detection, treatment, and vector control. Case detection and management was based on passive surveillance at fixed diagnostic and treatment facilities and on active screening of the population by mobile teams [8]. Primarily, screening consisted of testing entire populations using the card agglutination test for trypanosomiasis (CATT) by mobile teams, or of people presenting themselves for consultation at various health facilities [5,8]. All HAT cases were treated in health centers that were responsible for treatment and follow-up examinations. Patients in the early stage of the disease without involvement of the neurological system were treated with pentamidine, whereas all late-stage cases were treated with melarsoprol using established protocols [8]. However, in certain foci, refractoriness to melarsoprol was recorded in up to 25% of treated cases. When available, DFMO (difluoromethyl-ornithine, or eflornithine) was used to treat patients that were refractory to melarsoprol [8]. Tsetse fly (vector) control was conducted through intense and consistent trapping using monoconical or Lancien traps in targeted areas, with active community participation. Angola has been a strong proponent of the Pan African Tsetse and Trypanosomiasis Eradication Campaign (PATTEC) initiative and one of the few countries that continues to mobilize internal/local resources to enhance and sustain tsetse and trypanosomiasis control activities in the country [18].

### Factors that have contributed to recurrent outbreaks of HAT in Angola

Published records indicate that only 3 new cases of HAT were reported in Angola in the period just before the country gained its independence in 1975 [13]. However, this near elimination status of HAT did not last long before resurgence occurred. The main factor that contributed to the return of transmission was an armed civil conflict that lasted 27 years, severely disrupting and compromising sustainable implementation of disease control activities. Conflict has been identified as a crucial determinant of sleeping sickness outbreaks in sub-Saharan Africa [19]. In the case of Angola, the conflict led to massive migration of populations from province to province, in most cases through tsetse infested habitats, hence exposing people to tsetse bites and increased risk of acquiring HAT. Additionally, infected people, the most important reservoirs of the disease, could not access diagnostic and treatment centers due to insecurity and landmines. Surveillance activities were disrupted due to restricted movement of mobile teams to the villages in the endemic foci. Above all, the war exerted tremendous pressure on the national budget, and disease (including HAT) control was no longer a priority. Healthcare personnel were also heavily demotivated due to rising inflation [8]. HAT control activities were restored only after the signing of a UN-mediated peace agreement in 2002.

More recently, HAT surveillance and vector control activities were disrupted in Angola (probably more than in other HAT endemic countries) by the COVID-19 pandemic, leading to uncontrolled disease transmission. This was demonstrated by a sharp increase in number of new *g*HAT cases, from 33 in 2020 to 174 in 2021 [20]. It has been predicted through modeling of worst case scenarios on the impact of COVID-19 on HAT that disruption of active and passive surveillance could delay achieving the goal of eliminating transmission of the disease by 2 to 3 years [21].

Another factor that could have contributed to sustained transmission of *g*HAT is the presence of animal reservoirs. Whereas humans are the major reservoirs of *T*. *b*. *gambiens*e infections, domestic animals (i.e., pigs, small ruminants, and dogs) might also be involved in disease transmission [22]. However, the role of animal reservoirs in sustaining *g*HAT transmission is considered rather minor [23], with a recent modeling study in the D.R. Congo indicating that animal reservoirs alone are not able to maintain *g*HAT transmission [24]. Interestingly, the same study showed that the existence of animal transmission could delay the achievement of elimination of transmission goal in some settings [24]. Fortunately, the modeling study demonstrated that the likelihood of achieving elimination of transmission of *g*HAT by 2030 increases 6-fold when medical interventions are combined with vector control, even with animal transmission included in the model [24].

### Strategies to accelerate progress towards elimination of HAT in Angola

The government of Angola, through the Institute for Combating and Controlling Trypanosomiases (ICCT), the body mandated to control HAT under supervision of the Ministry of Health, aims to eliminate sleeping sickness in the country by 2030. In 2015, Angola introduced novel strategies to eliminate HAT, as part of a transboundary project with the DRC and RoC. The strategies implemented in the last 7 years to accelerate progress towards elimination of HAT as a public health problem include: (a) expansion of active and passive screening and expansion of the HAT diagnostic algorithm (b) raising community awareness about sleeping sickness; (c) strengthening vector control activities in endemic provinces; and (d) establishing and/or strengthening international partnerships and introducing new treatments. These strategies are further described below.

### (a) Expansion of passive and active screening and expansion of the HAT diagnostic algorithm

After the signing of a peace agreement in 2002, the Angolan MOH reactivated HAT control activities, coordinated by the ICCT. Active and passive screenings have been key pillars in the national HAT control strategy. Active screening is conducted on populations that are at risk of infection using the CATT test, whereas passive screening is conducted on suspected cases at the health facility using HAT RDTs. If either CATT or RDT is positive, a parasitological test, either the Capillary Tube Centrifugation (CTC) or mini-anion exchange centrifugation technique (mAECT) is conducted. A HAT case is confirmed if a parasitological test is positive. However, if a sample is CATT/RDT positive but negative by microscopy, it is subjected to a molecular test known as LAMP (loop-mediated isothermal amplification) or trypanolysis test. If LAMP/trypanolysis is positive, the person is considered a strong suspect and parasitological testing is repeated. This robust algorithm ensures that few or no HAT cases go undiagnosed. Treatment is only administered on the basis of a positive parasitological test. In addition to active and passive screening, reactive screening by small mobile teams (5 people) using cars is conducted in provinces where HAT cases have recently been identified. The mobile teams conduct door-to-door screening using HAT RDTs and test all RDT positive individuals by microscopy, including CTC and mAECT.

**Fig 4:**
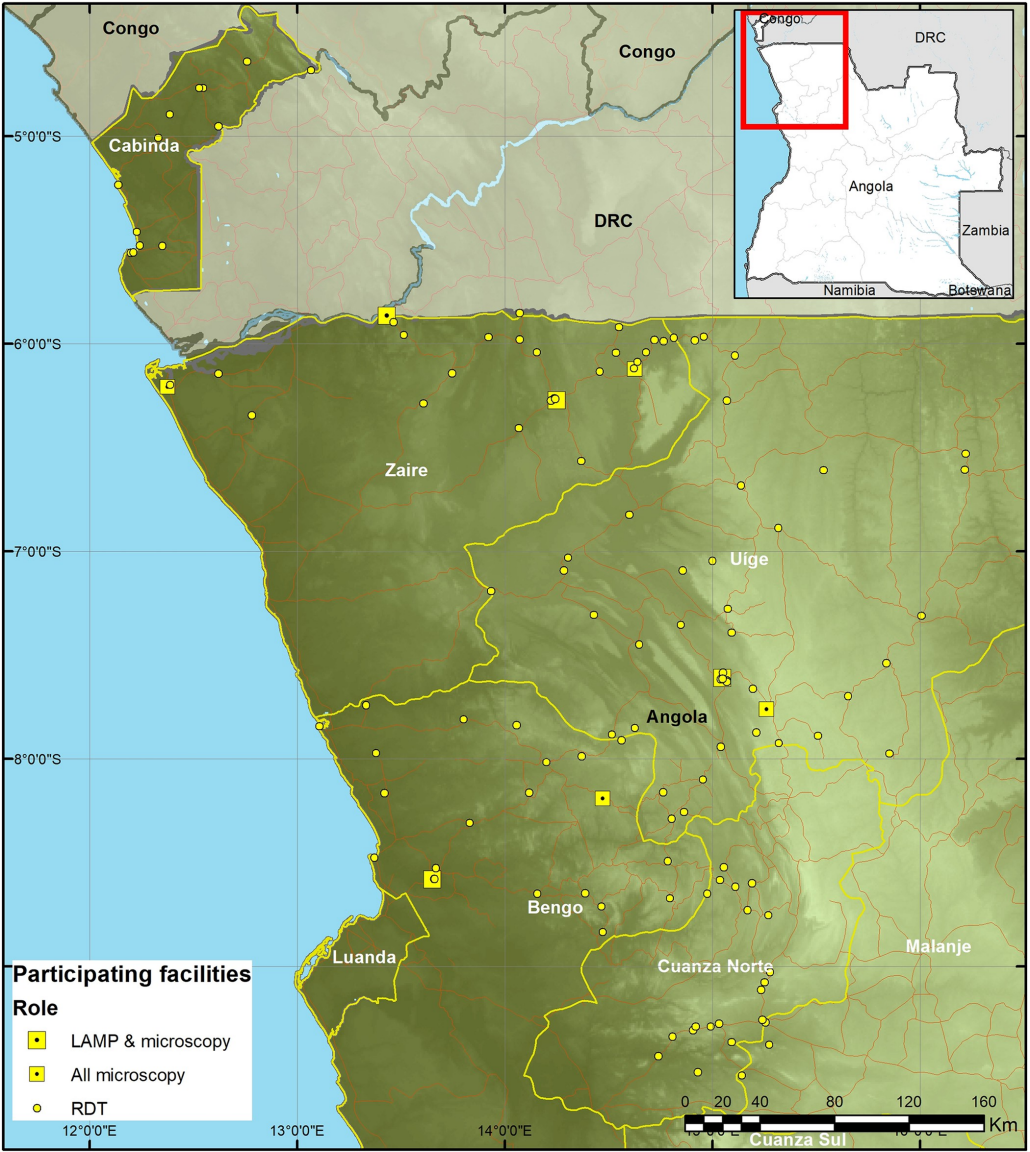
Map of Angola showing health facilities that were screening for HAT and the diagnostic tests implemented between 2017 and 2023. Map created with ESRI ArcPro v3.3. Boundary data from GADM (https://gadm.org/license.html), background elevation data is NASA SRTM30.

Since 2015, the Angolan MOH, working with partners, has prioritized integration of screening and testing for HAT at primary healthcare and community levels, raising awareness on the disease among healthcare workers and communities in the endemic areas, and supplementing these efforts with vector control. With funding support from DFID (United Kingdom’s Department of International Development, currently Foreign, Commonwealth and Development Office–FCDO), SDC (Swiss Agency for Development and Cooperation), and more recently Canton of Geneva, FIND has supported ICCT to intensify and roll out (in a phased approach) HAT screening and testing using RDTs. Roll-out of screening using RDTs was started in August 2016 in 2 provinces (Cabinda and Zaire) where by December 2017, 51 health facilities had been trained and equipped with the tests. Between January 2018 and July 2019, the roll-out was extended to 2 additional provinces (Bengo and Uige), increasing the number of health facilities screening for HAT using RDTs to 118. Thereafter, the roll-out continued progressively, and by December 2022, 157 health facilities, spread across all the 7 HAT endemic provinces, were screening for HAT using the test. Fig 4 shows health facilities that were screening for HAT and the diagnostic tests implemented in Angola from 2017 to 2023.

Among the facilities screening for HAT using RDTs are some that are strategically located and equipped to conduct confirmatory parasitological and/or molecular (LAMP) tests. From 2023, support for the intensification of screening for HAT has been through the Trypa-NO partnership funded by the Bill and Melinda Gates Foundation (BMGF). This partnership brings together FIND, Liverpool School of Tropical Medicine (LSTM), and the French Institute for Research and Development (IRD).

Considering that HAT is a transboundary disease, and to prevent the introduction of imported cases, the Angolan government has prioritized cross-border collaboration with the DRC and RoC. From 2014, the 3 countries have been implementing a collaborative project in the transboundary region of Kongo Central, coordinated by FIND. Project activities include characterization of health facilities, capacity building, upgrading of health facilities, and introduction of novel screening and diagnostic tools such as RDTs [18].

### (b) Raising community awareness about sleeping sickness

As the number of HAT cases continues to decline, perceptions of disease risk, early recognition of symptoms, and behavior of communities in endemic areas to take preventive measures and seek treatment is likely to wane. Therefore, sensitization of health workers and communities in endemic regions is essential. In Angola, this is being done through radio spots, print media, during active screening campaigns, and through strategically placed posters.

### (c) Strengthening vector control activities

**Fig 5:**
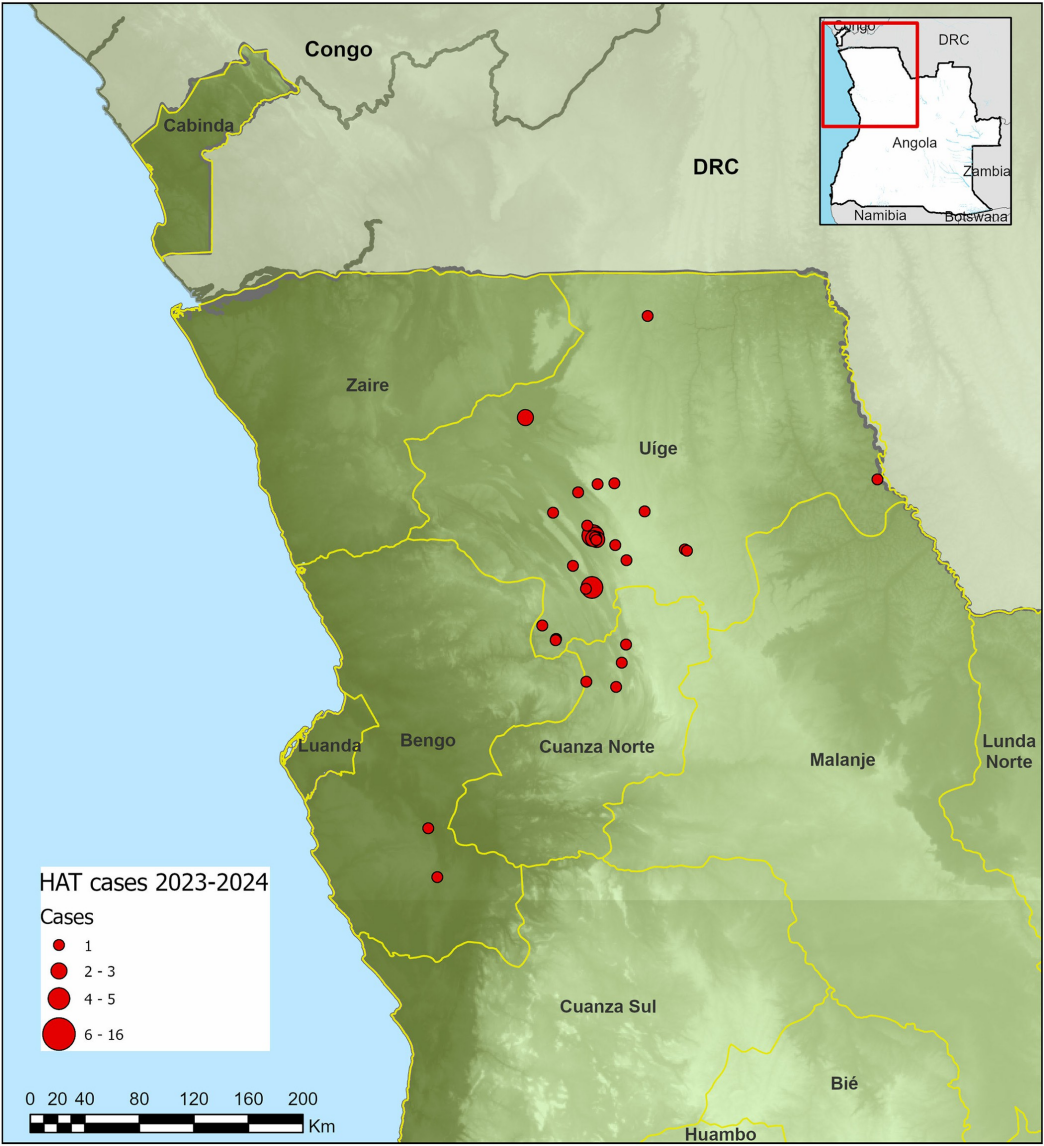
gHAT cases reported in Angola from 2023 to 2024. Map created with ESRI ArcPro v3.3. Boundary data from GADM (https://gadm.org/license.html), background elevation data is NASA SRTM30. It was not possible to map all cases but the footprint remains unchanged.

Vector control contributes to curbing transmission by reducing tsetse–human contact. In the context of HAT elimination in Angola, vector control activities are targeted/focused, escalated, or scaled back as needed. Out of the 18 provinces in Angola, tsetse flies are present in 14 [2], and 7 of these are endemic for gHAT. The endemic provinces are located in the north-western part of the country (Fig 1), with most recent cases (2023 to 2024) reported from the provinces of Bengo, Cuanza Norte, and Uige (Fig 5). Vector control activities against gHAT are carried out by the ICCT under the supervision of the MOH. Integrated vector control against tsetse and mosquitoes is also being introduced in collaboration with the malaria control program, and in particular, in collaboration with technical staff at the level of health districts. Collaboration is also being pursued with the Ministry of Agriculture, particularly with the institutes of veterinary service and of veterinary investigation, with a view to tackling the One-Health dimensions of disease transmission (such as trypanosome infections in pigs). Supporting institutions include the Angolan Armed Forces and Fundanga, a national nongovernmental organization [25]. Funding for vector control in Angola has until now been solely provided by the national government, demonstrating political goodwill and commitment. The country has been an active proponent of and player in the PATTEC initiative. The PATTEC initiative was established following a decision by African Heads of State and Government at the African Union (AU) Summit held in Lomé, Togo, in July 2000 (Decision AGH/Dec.156-XXXVI) and tasked with initiating a campaign to eradicate the threat of trypanosomiasis and tsetse flies from the African continent. As a result, an action plan was drawn up to achieve this objective. In Angola, the Ministries of Health and Agriculture led the implementation of the PATTEC initiative. Within the scope of the PATTEC initiative, the region of Luiana in the province of Cuando Cubango, *Glossina morsitans centralis* was targeted for control through aerial and ground spraying in 2009. Additionally, impregnated pyramidal traps were used to control tsetse flies in the provinces of Cuanza Norte, Uíge, Zaire, Malange, Cuanza Sul, Luanda, and Bengo.

Indeed, impregnated pyramidal traps and ground spraying continue to be key interventions for controlling tsetse flies in gHAT endemic areas of Angola. Traps are deployed first, and tsetse flies are collected after approximately 1 week. Where high densities of flies are detected, ground spraying follows. Local communities, and where present the armed forces, are engaged in field activities, both in the deployment of traps and the collection of captured tsetse flies. The activities of the ICCT teams normally take place over a period of 20 to 30 days. Thereafter, traps are maintained and checked by local communities for periods of up to 6 months [25]. Information on the impact of vector control on tsetse densities is patchy and comprehensive data are lacking. However, a sizable and positive impact is reported in some zones, while in other areas, more limited and short-lived impacts are reported. The ICCT plans to intensify vector control in the gHAT endemic areas, and to extend it to non-endemic areas, mostly with funding from the national government. Plans are also underway by ICCT to introduce new vector control tools such as “Tiny Targets,” insecticide-impregnated panels made of material that attract and kill tsetse flies [26], and to strengthen entomological capacity, including for xenomonitoring as part of the Trypa-NO! partnership.

### (d) Establishing and/or strengthening international partnerships and introducing new HAT treatments

Whereas HAT control in Angola has been, and is largely resourced by the national government, partnerships have been a critical pillar in the fight against sleeping sickness in the country. In the early days, the ICCT partnered with the United Nations High Commissioner for Refugees (UNHCR), Caritas de Angola, Norwegian People’s Aid, Médecins Sans Frontières (MSF), Belgian Technical Cooperation (CTB), Swiss Tropical and Public Health Institute (Swiss-TPH), Institut de Recherche pour le Développement (IRD, France) and WHO, to drive HAT control in Angola. Areas of focus in these earlier partnerships included: setting up systematic HAT screening systems to detect cases in the early stages (at stage 1), establishing mobile diagnostic laboratories and treatment clinics/centers, and conducting research to discover shorter-term and more effective ways to use the highly toxic drugs used to treat the advanced form of the disease known as stage 2 gHAT [27].

More recently, international partnerships have been forged to accelerate progress towards the elimination of gHAT as a public health problem in Angola. An example is the Trypa-NO! partnership [28] that is driving elimination of HAT by harmonizing and integrating screening, diagnosis, and treatment of HAT cases with tsetse fly control. Additionally, the nonprofit organization, Drugs for Neglected Tropical Diseases (DNDi) has recently introduced fexinidazole in Angola, a breakthrough medication for gHAT. Fexinidazole is effective in the treatment of both the blood stage and non-severe stage 2 gHAT, making invasive procedures of stage determination unnecessary in most cases [29]. Before the introduction of Fexinidazole in 2022, Angola was using Pentamidine and Eflornithine for the treatment of HAT stages 1 and 2, respectively. Whereas, Pentamidine is still being used, the use of Eflornithine as a single molecule was stopped in 2013. However, from 2013, the country started using Nifurtimox-eflornithine combination therapy (NECT) for treating HAT stage 2. Drugs for treatment of HAT are provided at no cost by WHO, which has also been supporting some active screening and capacity building activities.

### Key achievements in the push towards elimination of gHAT in Angola

Table 1 shows the overall results of HAT screening activities in Angola between 2016 and 2023. Overall, 450 gHAT cases were identified, from a total of 493,796 people screened using different strategies over this period, translating to an overall positivity rate of 0.09%. The largest proportion of people screened (72.5%) and cases identified (60.9%) were through active screening. Approximately 26% of the total number of people screened were by passive surveillance, which identified 37.8% of the cases. The number of people tested through passive surveillance varied from year to year, and was attributed to irregular use of HAT RDTs in health facilities located in endemic municipalities, and to weaknesses in stock management. Between 2014 and 2022, the number of HAT cases reported annually has been consistently below 50, except in 2018 (*n* = 79 cases) and 2021 (*n* = 174 cases). The increase in number of reported cases in 2018 coincided with the roll-out of the intensive passive surveillance program, which improved coverage of the population at risk. However, the relatively high number of cases reported in 2021 could be attributed to either the intensified passive surveillance, the impact of COVID-19 in 2020 that disrupted surveillance and treatment (leading to uncontrolled transmission), or to a combination of both factors. Given that the majority of these 2021 cases were recorded in the Bolongongo/Banga focus, which was considered a new gHAT focus, intensive passive surveillance was most likely the main driver of the increase in number of cases in that year. Door to door reactive screening only started in 2023, as an added strategy for intensified case detection, as the push towards gHAT elimination gains momentum in Angola. Over the last 2 years (2023 to 2024), new gHAT cases have only been reported in 3 of the 7 endemic provinces (Fig 5), demonstrating shrinkage in sleeping sickness foci in Angola.

**Table 1:** Number of people screened for g-HAT using different strategies and cases identified in Angola from 2016 to 2023.

	HAT screening strategy							
	Active screening		Reactive screening		Passive screening		Total	
Year	No. tested	No. HAT cases	No. tested	No. HAT cases	No. tested	No. HAT cases	No. tested	No. HAT cases
2016	1,065	0	-	-	25,405	20	26,470	20
2017	31,897	3	-	-	10,741	15	42,638	18
2018	102,758	48	-	-	15,301	31	118,059	79
2019	88,718	10	-	-	24,694	20	113,412	30
2020	1,865	7	-	-	21,500	26	23,365	33
2021	47,965	151	-	-	7,256	23	55,221	174
2022	50,066	25	-	-	5,221	19	55,287	44
2023	33,643	30	4,521	6	21,180	16	59,344	52
**Total**	**357,977**	**274**	**4,521**	**6**	**131,298**	**170**	**493,796**	**450**

### Health system supporting HAT control in Angola

In 2015, Angola published its National Health Sanitary Development Plan (NHSDP) 2012–2025. This is a key policy, operational and strategic document that is designed to support the national health system technically, financially, and politically. The document contributes to the realization of the country’s long-term strategy (Angola 2025) and feeds into the national health policy. Apart from the NHSDP, the country also developed the National Health Development Plan (NHDP) 2012–2025, which recognizes health as the primary factor for overall development, and underscores the government’s continued commitment to fighting poverty and promoting sustainable improvement of the health of its people. One of the nine (9) programs under NHDP is prevention and control of endemic diseases such as HAT and other parasitic infections [30]. Hence, a policy framework for HAT control and elimination exists, and there is commitment at the highest level of government.

The structure of the Ministry of Health (MoH) in Angola is hierarchical, consisting of 3 levels of health administration; i.e., national, provincial, and municipal. The national level includes Cabinets of the Minister and Secretaries of State, Support Boards, and Central Executive Boards. The provincial level is made up of Provincial Health Offices that depend administratively on provincial governments and receive technical guidance from the national level. Financial support for implementation comes from both national and provincial budgets. At the municipal level, the Municipal Health Directorates depend on the Municipal Administration for administrative issues, while implementation guidance comes from the provincial and national levels. Overall, there are 20 central and specialty (national) hospitals, 125 provincial general hospitals, and 165 municipal hospitals. This hierarchical structure facilitates systematic implementation of HAT control/elimination programs.

A unique aspect of Angola’s health system is that the country’s health budget is not as donor-dependent as those of other sub-Saharan African countries. The government’s contribution accounts for approximately 70% of the total health expenditure, with the rest coming from private sources, especially out-of-pocket [30]. This internal funding of health programs has been a key driver to the success of HAT control in Angola.

## Conclusion and future outlook

It has been more than a century since the first case of HAT was reported in Angola. Over this period, significant progress has been made in the fight against the disease. Control strategies put in place by the colonial administration (active case detection, mass treatment of populations, and follow ups) from 1920s to early 1970s led to significant reduction in HAT cases, to the extent that at independence in 1975, HAT was not considered a public health problem in Angola, with only 3 HAT cases reported in 1974. However, this near elimination status was severely affected by an armed civil conflict that lasted nearly 30 years and significantly disrupted HAT control activities. Intermittent support by international partners and nongovernmental organizations was crucial in sustaining control activities during the conflict, albeit at limited and non-programmatic levels. The commitment of the Angolan government to fund most HAT control activities is a lesson that other HAT endemic countries could learn from. Leveraging the power of international partnerships in disease control/elimination is a key lesson learned from this case study. Since 2014, owing partly to the adoption and intensive roll out of new technologies (e.g., RDTs), renewed political commitment, external support, and partnerships (e.g., Trypa-NO!), there has been a renewed period of coordinated HAT control with the goal of advancing to elimination. However, the results of these efforts are mixed and difficult to fully interpret at present, given that the number of cases being reported by the national HAT control program has been fluctuating and cases are being reported in new areas. Between 2014 and 2022, the number of cases reported annually in Angola has been consistently below 50, except in 2018 (*n* = 79 cases), 2021 (*n* = 174 cases), and 2023 (*n* = 52 cases). Considering the relatively low number of cases recorded annually during the last 7 years, there is renewed hope that Angola could soon begin to progress towards gHAT elimination. However, factors contributing to the fluctuation in the number of new reported cases need to be identified and addressed at programmatic level.

The government of Angola together with its partners will, in addition to ongoing activities, need to prioritize strengthening vector control as one of the key strategies in achieving the elimination goal. This should include widespread deployment of innovative tools such as Tiny Targets, and training of personnel and communities on deployment and monitoring the efficacy of such tools. Sustaining and strengthening of surveillance in endemic border regions should also be prioritized. Although efforts have been made to have cross-border collaboration between Angola, the DRC, and RoC, such efforts have only concentrated in the Kongo Central region. These efforts should be expanded to cover the expansive borders between Angola, RoC, and DRC where the disease is endemic. As elimination becomes more and more apparent and recent reports indicating shrinkage of sleeping sickness foci, Angola should start preparing a technical dossier to WHO on the elimination of gHAT in the country, for it to be validated as having eliminated the disease as a public health problem.
